# Myocardial Regenerative Properties of Macrophage Populations and Stem Cells

**DOI:** 10.1007/s12265-012-9383-6

**Published:** 2012-06-09

**Authors:** Maria Paola Santini, Nadia Rosenthal

**Affiliations:** 1 National Heart and Lung Institute, Imperial College London, Harefield, UK; 2 Australian Regenerative Medicine Institute, ARMI, Melbourne, Australia; 3 Heart Science Centre, Hill End Road, UB9 6JH Harefield, UK

**Keywords:** Cardiac progenitor cells, Macrophage populations, Cardiac regeneration, Fibrosis, Inflammation

## Abstract

The capacity to regenerate damaged tissue and appendages is lost to some extent in higher vertebrates such as mammals, which form a scar tissue at the expenses of tissue reconstitution and functionality. Whereas this process can protect from further damage and elicit fast healing, it can lead to functional deterioration in organs such as the heart. Based on the analyses performed in the last years, stem cell therapies may not be sufficient to induce cardiac regeneration and additional approaches are required to overcome scar formation. Among these, the immune cells and their humoral response have become a key parameter in regenerative processes. In this review, we will describe the recent findings on the possible therapeutical use of progenitor and immune cells to rescue a damaged heart.

## Introduction

Regeneration of organs and appendages in lower vertebrates is a process that involves several molecular and cellular mediators including bioelectrical signals, thrombin activation, immuno-modulation, wound epithelium formation and stem cell activation. In mammals, tissues such as liver, skin and endometrium can regenerate, presumably due to their normally high turnover rate. Rare examples of mammalian appendage regeneration include seasonal deer antler growth [1]. These regenerative properties account for cell cycle re-entry (liver), stem cell differentiation (skin) and wound epithelium formation (deer antler growth), resembling processes observed in lower vertebrates.

An emerging concept is that the mammalian heart also retains regenerative potential reminiscent of lower vertebrate cardiac repair, but the cardiac regenerative response might be insufficient to repair the extensive damage caused by myocardial infarct (MI), the most common type of cardiac insult in humans. Although resident cardiac stem cells have been reported to replenish the injured myocardium [2], a complete functional recovery after damage is not achieved.

A recent report suggests that the fully regenerative mechanisms in zebrafish hearts, which are mediated by cardiomyocyte proliferation, are latent in mammalian heart. Indeed, a murine model resembling zebrafish heart injury showed that resection of the left ventricular apex in 1-day-old neonatal mice induces cardiomyocyte proliferation and full heart regeneration in 8 weeks [3]. Notably, the proliferative response was lost in 7-day-old mice, coinciding with cardiomyocytes binucleation and increased cardiac workload.

Cardiomyocyte turnover in adult humans is a rare event, as measured by the level of ^14^C in proliferating cardiac cells making use of the atmospheric increased of this isotope as consequence of nuclear weapons’ testing during the Cold War. The analysis showed that only 45 % of cardiomyocytes would be predicted to be renewed over a normal human lifespan [4]. Nonetheless, these studies leave open the formal possibility that the processes of regeneration mediated by cardiomyocytes proliferation could be re-habilitated after injury, by reversing the mechanisms that block cell-cycle re-entry in a controlled therapeutic setting.

Two additional prerequisites must be satisfied for successful regeneration of the cardiac ischaemic region. Coronary arterioles and capillary structures must be formed in order to bridge the dead tissue and establish communication with the normally perfused vessels of the viable myocardium. Additionally, the new vascular supply has to permeate the myocyte bed to preserve their survival, and favour their growth, differentiation and contractile function.

In mammals, the presence of a circulating cell population that could restore cardiac vasculature has gained credibility from observations that CD34-positive cells from human peripheral blood localize to areas of neovascularization when injected into nude mice with hindlimb ischemia [5]. The dearth of information regarding the precise origin and function of circulating endothelial progenitor cells (EPCs) has prompted animal experiments in which stem cells isolated from bone marrow [6–8] or peripheral blood [9] and enriched for various surface markers were analysed for their regenerative properties to restore cardiac function and form new blood vessels. Despite these initial analyses, subsequent studies in animals with reconstituted genetically marked bone marrow (LacZ, GFP) concluded that bone marrow-derived cells were detectable only as perivascular cells supporting the growth of new vessels but were not integrated in the forming vessels [10, 11]. Thus, it appears that the majority of endothelial-like cells in the peripheral blood are monocytes that activate resident EPCs to form vessels by releasing a plethora of pro-angiogenic factors.

Based on emerging evidence for immune engagement in regeneration, here we propose novel ways in which combinatorial stem cell treatment and modulation of the immune cells may have important therapeutic and pharmacological implications for clinical treatment of heart disease.

## Stem Cells for Cardiac Regeneration

Reminiscent of developmental programs, adult mammalian heart seems to retain stem cell populations capable of multipotent differentiation. These cells have been variously described as different populations by the expression of specific cell surface markers (c-kit, Sca-1 and MDR-1).

In recent years, numerous studies have aimed to determine the efficiency of those stem cells to regenerate the heart via cell therapy combined with tempering the hostile environment of the infarct by administration of cell survival and anti-inflammatory molecules. Important advances in the control of stem cell fate have also moved the field of regenerative medicine closer towards applicable therapies for cardiac muscle regeneration. However, significant obstacles must still be overcome, including the incomplete cell differentiation of stem cells (with the associated danger of tumour formation), the paucity of organ-specific stem cell resources (specifically in adult/aged organs) and the immunogenicity of the transplanted cells (graft rejection).

### Cardiac Stem Cells

The earliest cardiovascular progenitors to be traced and studied during embryogenesis express the transcription factor Islet 1 (Isl1) [12]. *Isl1*-null hearts failed to undergo looping morphogenesis and appeared to have a common atrium and a uni-ventricular chamber, whereas the right ventricle and the outflow tract were absent [13]. These observations suggest that Isl1 is expressed in cells of the second heart field, which contribute to both the venous and arterial poles of the heart [12]. Interestingly, Prall and co-workers report that, in contrast to Isl1 mRNA, Isl1 protein is expressed at E7.5 throughout the anterior intra-embryonic coelomic walls and proximal head mesenchyme, regions that encompass both first and second heart fields in mouse [14]. Similarly, during neurula stages in Xenopus, Isl1 is co-expressed with Nkx2-5 throughout the cardiac crescent, which is the first heart field in amphibians [15]. These data suggest that Isl1 might be a pan-cardiac progenitor marker, but additional work is needed to clarify this issue.

Using conditional genetic marking techniques in the mouse, Laugwitz and co-authors performed Cre-recombinase-triggered cell lineage tracing experiments to irreversibly mark isl1-expressing cells as well as their differentiated progeny during embryonic development [16]. Isl1-IRES-Cre mice were crossed into the conditional Cre reporter strain R26R, in which Cre-mediated removal of a stop sequence results in the ubiquitous expression of the lacZ gene under the control of the endogenous Rosa26 promoter [16]. The analysis showed that Isl1+ progenitors make a remarkable contribution to the cells of the conduction system, primarily to the sino-atrial node. Additionally, galactosidase positive staining has been observed throughout the proximal aorta, the trunk of the pulmonary artery and the stems of the main left and right coronary arteries [16]. The co-expression of lacZ with endothelial and smooth muscle-specific markers, such as CD31 (Pecam1), VE-cadherin (cadherin 5) and smooth muscle myosin heavy chain (SM-MHC; also known as myosin heavy chain 11), revealed that Isl1+ precursors can give rise to vascular lineages [16].

In vitro, by tamoxifen inducible Cre-lox technology, postnatal Isl1+ cell population and its progeny have been selectively marked at a defined time and purified to relative homogeneity [16]. The ability of these cells to self-renew on a cardiac mesenchymal feeder layer and to be stimulated to differentiate into fully mature functional cardiomyocytes indicates that they represent native cardiac progenitors, remnants of the embryonic Isl1+ precursors [16]. Recently, the presence of Isl1 cells in the adult murine heart has been identified as clusters in the inter-atrial septum, scattered in the wall of the great vessels, and as a delimited cluster between the right atrium and the superior vena cava [17]. Notably, in this report the authors observed that Isl1+ cell number and localization remained unchanged between 1 to 18 months of age [17]. Furthermore, these cells were not present at the infarcted area 28 days after myocardial infarct induction [17], suggesting that that Isl1-positive cells are not a source of cardiac progenitors.

Recently, cardiac stem cells (CSCs) residing in the epicardium have received considerable attention. This outer layer of cells surrounding the myocardial tissue has been shown to have an important role in regeneration of zebrafish [18] and of the mammalian heart [19, 20]. These studies reported that at least a subset of Sca-1+ cells originally shown to reside in the murine heart are of epicardial origin and can differentiate into all cardiac cell types, highlighting the importance of the epicardium in mammalian cardiac stem cell-mediated repair.

In the first study [20], activated adult progenitors were shown to re-express a key embryonic epicardial gene, Wilm’s tumour 1 (Wt1), after myocardial injury. Furthermore, these cells are positive for Sca-1 and give rise to de novo cardiomyocytes that structurally and functionally integrate with resident muscle after treatment with thymosin β4 [20].

In the second report [19], a subpopulation expressing the platelet-derived growth factor receptor alpha was found amongst the cells positive for Wt1 and Sca-1, occupying a perivascular niche and showing broad trans-germ layer potency in vitro and in vivo [19].

Epicardial cells with the potential to transdifferentiate into other cell lineages have been observed also in human fetal and adult hearts [21, 22]. Limana and co-authors showed that c-kit+ and CD34+ cells can be detected in human fetal and adult epicardium as two distinct populations capable of giving rise to myocardial precursors and vascular cells in vitro [21]. The authors further showed that, in a mouse model of myocardial infarction, there was an increase in c-kit+ cells in the subepicardial space. Interestingly, 3 days after infarction these cells expressed GATA4, whereas at 1 week c-kit+ cells co-expressed endothelial or smooth muscle cell markers [21]. In another study, it has been observed that in vitro culture of primary human epicardial cells present characteristics of smooth muscle cells (SMCs) if treated with transforming growth factor-beta or bone morphogenetic protein 2 (BMP2) [22].

Although promising in their possible therapeutic applications, these studies require more experimental analyses to determine the efficiency and efficacy of epicardial cells to regenerate the heart after injury and to differentiate in different lineages in vitro.

**Fig. 1:**
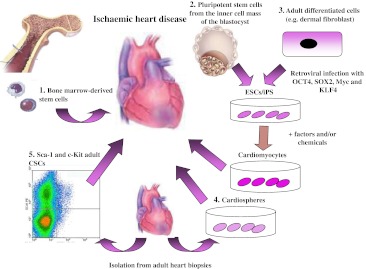
Stem cell therapies to heal infarcted heart. Different stem cells have been used to recover cardiac function after ischaemic diseases. Bone marrow-derived stem cells (*1*), c-kit adult cardiac stem cells (*5*, CSCs) and cardiospheres (*4*) have been employed in clinical trials with promising results. Immunogeneicity and ethical problems obstruct the usage of ESCs and iPSCs (*2*, *3*) for clinical studies. The limits and potentialities of Wt1- Sca-1-positive epicardial cells have not been exploited

So far, the most characterized adult progenitor cells in the heart are the Lin−/c-kit+ cells. These cells are self-renewing, clonogenic, multipotent in vitro and in vivo, and give rise to myocytes, smooth muscle and endothelial vascular cells (Fig. 1) [23].When injected into an ischaemic rat heart, a population of these cells or their clonal progeny reconstitutes large portions of the injured myocardial wall [23]. The regenerated myocardium contains small myocytes that present the anatomical, biochemical and functional properties of young myocytes [23]. Recent evidence shows that Lin−/c-kit+ cells also have the ability to form conductive and intermediate-sized coronary arteries when injected in infarcted rat heart [24]. Resistance arterioles and capillaries are connected with primary coronary circulation, indicating that Lin−/c-kit+ cells have a dual role in vessel and contractile muscle formation [24].

Importantly, not all studies recognize c-kit-positive cardiac progenitor cells as in vivo promise for therapeutic cardiac regeneration. Indeed, using transgenic reporter mice and through genetic read-outs for lineage tracing, c-kit cells did not differentiate in cardiomyocytes in vivo after transplantation, questioning their claimed efficiency in regenerating the rodent hearts [25]. Interestingly, Zaruba and co-authors used double transgenic beta actin-green fluorescent/alpha Myosin Heavy Chain-nuclear beta galactosidase mice (ACT-EGFP/MHC-nLAC) to show that c-kit+ cardiac cells from neonatal hearts acquired a cardiomyogenic phenotype in coculture with fetal mouse cardiomyocytes, independently of cell fusion [26]. This activity was diminished in c-kit+ cells isolated from adult hearts [26]. Moreover, c-kit+ cells from adult hearts failed to undergo cardiomyogenic differentiation when transplanted into infarcted adult hearts, indicating that the ability of cardiac specific c-kit+ cells to acquire a cardiomyogenic phenotype is subject to temporal limitations or that the cardiomyogenic population is lost [26]. Interestingly, it has been identified a population of c-kit+ cells in human atrial biopsies that could be subdivided based on the expression of CD45 [27]. The c-kit+CD45− cells coexpressed endothelial cell markers such as CD31, CD34 and FLK-1, whereas the c-kit+CD45+ population was determined to be of mast cell identity [27]. However intriguing, further characterization of the above human populations should be supported by ex vivo expansion and differentiation studies, as well as cell transplantation analyses, to rule out their efficiency in cardiac regeneration.

In addition to the above populations, cells growing into spherical structures termed cardiospheres [28] are recognized as a distinct adult progenitor cell population.

Cardiospheres are a heterogeneous population of cells obtained from adult hearts (e.g. from an endomyocardial biopsy) in culture (Fig. 1). These cells form a three-dimensional spherical structure with high proliferative rate and capable of forming differentiated contractile cardiomyocytes [28, 29]. Transplanted in murine [28] and rat [30] models of myocardial infarct, cardiospheres improved left ventricular function and decreased scar size.

Interestingly, it has been shown that allogeneic transplantation of cardiospheres in a rat myocardial infarct model without immunosuppression is safe, promotes cardiac regeneration and improves heart function, mainly through stimulation of endogenous repair mechanisms [30]. The indirect mechanism of action suggests the persistence of benefit after removal of the transplanted cells by the host immune system and has motivated the testing of allogeneic cardiospheres in humans (Fig. 1) [31].

Hoechst exclusion cells, contained in a Verapamil-sensitive side population, have been isolated from adult murine hearts, showing spontaneous beating areas if treated with oxytocin or trichostatin A [32]. In vivo, they are present in the perivascular area and upon injury differentiate in cardiomyocytes, endothelial and smooth muscle cells in a percentage of 4.4, 6.7 and 29 %, respectively [32]. Their therapeutic potential has yet to be investigated making this population not as fully exploited as others described above.

Although resident cardiac progenitor cell populations have now been identified, the insufficiency of endogenous stem cells to alleviate acute and chronic damage to mammalian cardiac tissue remains to be overcome. Overall, the paucity in progenitor cell number in aged or diseased hearts makes difficult to expand them to replace the massive amount of cardiomyocytes lost during ischemia.

### Pluripotent Stem Cells

New research avenues involving exogenously administered pluripotent stem cell-derived cardiomyocytes have shown cardiac function improvement in animal models of myocardial repair, underscoring their therapeutic potential. Therefore, considerable emphasis has been placed on enhancing differentiation of these cells and exploiting their therapeutic use as a goal of regenerative medicine.

Pluripotent stem cells have the capacity to differentiate into cells derived from any of the three germ layers (Fig. 1). Embryonic stem cells (ESC) are pluripotent stem cells with the greatest potential to differentiate into cardiomyocytes and replace the damaged cardiac muscle. In vitro analysis showed that human ESC-derived cardiomyocytes spontaneously contract and generate action potentials of multiple cardiac phenotypes, as well as expression of specific cardiac protein and myofibrillar organization [33]. In vivo ESC-derived cardiomyocytes can function as pacemakers in models of atrioventricular-blocked hearts [34]. In an acute cardiac injury model, human ESC-derived cardiomyocytes formed gap junction and grafted cells behaved as a syncytium. Most studies report functional improvement as apparent as 4 weeks [35, 36], whereas one study extended the analysis up to 12 weeks in mice, in which the benefit of the injected cells was no longer present [37]. Although it cannot be ruled out that paracrine effects are responsible for the increased functional parameters, most likely differences in beating frequency may account for the failure to improve long-term cardiac functions in mouse models (murine heart beats 300–600 times per minute, while human cardiomyocytes beat 60–100 times per minute) [38].

The use of non-autologous human ESC in a therapeutic context is confounded by their inherent immunogenicity, although mixed chimaerism and reversible reactivation of thymic function by analogues of luteinizing-hormone-releasing hormone showed immune tolerance after transplantation of ESCs [39]. In practice, the advantages of using ESCs are often outweighed by ethical and political considerations, which constitute the chief impediments to the clinical application of these cells. Several strategies to overcome the use of human embryos involve the production of pluripotent stem cells from adult cells. Remarkably, retroviral-mediated overexpression of only four proteins (OCT4, SOX2, Myc and KLF4) in mouse embryonic or adult fibroblasts generated inducible pluripotent stem-cell-like cells (iPS) able to differentiate into derivatives of the three germ layers (Fig. 1) [40]. iPS cells are ideal candidates for autologous transplantation, since production of patient specific iPS cells can theoretically overcome the problem of rejection and immunosuppressive therapy.

However promising, both iPS and ESCs form teratomas if undifferentiated derivatives are present in the pool of injected cells. The carcinogenic nature of ESCs and iPS limits their therapeutic application and poses an important challenge. Furthermore, they form heterogeneous populations of cardiomyocytes in culture (atrial, nodal and ventricular), having different action potentials. Therefore, effective differentiation methods need to be developed to yield highly purified cell populations for transplantation to avoid arrhythmias and tumour formation.

Indeed, direct transdifferentiation of fibroblast in cardiomyocytes has been acclaimed as a safer and more practical step towards autologous cell therapy [41]. The combination of three transcription factors (MEF2C, GATA4, TBX5) activated the Myh6 promoter in murine cardiac fibroblasts. About 4 % of the cells expressed cardiac troponin T and 1 % showed spontaneous beating. Another attempt showed that by blocking the JAK–STAT pathways and by the addition of BMP4, 40 % of murine embryonic fibroblasts expressed Troponin T [42], indicating an increased efficiency in producing autologous cardiomyocytes.

Although encouraging results in tumour reduction have been obtained using methods to preselect for ESCs that differentiate along a cardiac lineage, or to preserve pluripotency in absence of Myc for iPS cells, a mere reduction in tumour incidence will not be sufficient to justify clinical trials.

### Bone Marrow and Mesenchymal Stem Cells

Considerable interest in bone marrow-derived cells (BMCs) was prompted by Orlic report describing a bone marrow stem cell population lineage negative (Lin−) and c-kit+ capable of transdifferentiating into cardiomyocytes and inducing regeneration of murine myocardial tissue after infarct [8]. In adult human hearts, the first evidence that adult BMCs participate in the formation of cardiomyocytes was based on reports of Y-chromosome-positive cardiomyocytes in female donor hearts transplanted in male recipients [43]. However promising, further studies in animal models of myocardial infarction showed that BMCs differentiated into cardiomyocytes after transplantation but at a very low rate [44]. In other analyses, it was shown that that BMCs do not form cardiomyocytes but instead become mature blood cells after transplantation [6, 45]. Furthermore, additional studies have indicated that BMCs are capable of fusing with cardiomyocytes, explaining the appearance of BMC-derived myocytes [7, 46]. Although no consensus exists on whether BMCs differentiate into cardiomyocytes in vivo, animal studies and clinical trials (described in the next paragraph) have shown improvements in ventricular function when BMCs are administered after infarction, implicating paracrine signalling as the major mechanism of action [47, 48].

Being BMCs composed of a heterogeneous cell population (haematopoietic stem cells, endothelial stem cells, multipotent adult progenitor cells, mesenchymal stem cells) [49], the beneficial outcomes may derive from one or a combination of several distinct subsets of cells. Among them, mesenchymal stem cells (MSCs) have received increasing interest in recent years for their immunoregulatory function and beneficial effects in healing damaged tissues.

MSCs were first identified in bone marrow [50] and recently have been found in adipose tissue [51]. These cells are multipotent and can differentiate into bones, cartilage and adipocytes [52].

In vitro approaches to determine their cardiac lineage commitment showed that MSCs from bone marrow, exposed to 5-azacytidine, differentiated into cardiomyocytes, exhibiting spontaneous action potential and mature sarcomeres [53]. Furthermore, human MSCs expressed cardiac protein in vitro by creating three-dimensional aggregates [54].

In clinical studies, MSC beneficial effects are well known. Indeed, these cells are used to treat secondary progressive multiple sclerosis (NCT01056471), chronic allograft nephropathy (NCT00659620) and as prevention for graft rejection and graft-versus-host disease (NCT00504803, NCT00972660, NCT00366145, NCT00284986).

In cardiovascular studies, the administration of MSCs to pig infarcts stimulated endogenous CPCs to contribute to repair processes [55]. In clinical trials, intracoronary administration of large numbers of autologous MSCs in patients with acute myocardial infarction (AMI) induced a 14 % improvement in ejection fraction [56]. Furthermore, allogeneic MSCs administered to patients intravenously within 10 days of infarction were well tolerated and were associated with decreased arrhythmias and an improvement in some indices of contractile function [57].

From the above studies, it is clear that the main function of MSCs is to elicit immunosuppression in inflammatory environments. Indeed, MSCs hold an immunoregulatory capacity by inhibiting T and B cells, as well as natural killer cells and dendritic cells [58]. Furthermore, MSCs are also immunoprivileged cells, due to the low expression of class II major histocompatibility complex [58], favouring therefore their usage in allogeneic transplantation studies.

In future analysis, it would be interesting to investigate whether a resident cardiac MSC population participate to cardiac repair by regulating inflammatory response and eliciting angiogenesis and remodelling after infarction.

### New Understanding in Cell Therapy and Cardiac Function Amelioration

Despite the lack of agreement in the beneficial outcomes of resident stem cells in regenerating infarcted hearts, the majority of studies in which BMCs were infused into infarcted murine hearts showed an improvement of global cardiac function and enhancement of blood flow (Fig. 1). Based on these results, human clinical trials were initiated in 2001 to treat patients with cardiac ischemia with autologous circulating blood or bone marrow-derived cells. The initial pilot studies (TOPCARE-AMI, BOOST trials) showed an improvement of global left ventricular ejection fraction by six to nine percentage points [59, 60]. These results were confirmed in a larger, double-blind, randomized trial called REPAIR-AMI. In this study, the global and regional ejection fraction in the bone marrow-treated group improved modestly but significantly by 2.9 % compared to the placebo-treated group. Notably, a larger study called ASTAMI trial did not show any benefit on the left ventricular functional parameters [61]. The two trials differed in the treatment of the isolated cells and this could have affected their functional capacity.

In a systematic review, Clifford and co-authors observed that BMCs treatment in acute myocardial infarct patients improved left ventricular ejection fraction over conventional therapies such as primary angioplasty in long-term follow-up analyses (60 months) [62]. Increased ejection fraction correlated with reduced infarct size and cardiac wall motion, although the incidence of mortality and morbidity was not statistically significant compared to conventional therapies [62].

Recently, two phase 1 trials showed promising results in cell therapy by employing autologous transplantation of cardiospheres [31] or c-kit-positive CSCs (Fig. 1) [63]. The study by Bolli and colleagues, Stem Cell Infusion in Patients with Ischemic Cardiomyopathy (SCIPIO), tested the safety and efficacy of c-kit+ CSCs for the treatment of ischaemic heart disease. The initial results showed that 1 year after intracoronary infusion of these cells, infarct size decreased by 30 % and ejection fraction increased 8 % after only 4 months. The second study by Makkar and colleagues, called Cardiosphere-Derived Autologous Stem Cells to Reverse Ventricular Dysfunction (CADUCEUS), showed that 6 months after intracoronary infusion of cardiospheres, regional contractility and viable heart mass increased, while scar mass decreased as assessed by magnetic resonance. These data correlated with survival of all patients receiving the cells and with lack of tumour formation, prompting the employment of the cells for further, large phase II clinical trials.

Promising as these studies may be, significant challenges remain to be overcome. Whereas c-kit+ CSCs infusion recover ejection fraction [63], cardiospheres transplantation improved local systolic function without affecting ejection fraction [31].

To shed light on these contradictory results, two reports showed that measurements of local function underlying decrease or increase of wall stresses after myocardial infarction and cell transplantation are more indicative than measurements of global function, such as ejection fraction and/or fractional shortening. The report by Williams et al. [64] shows that intramyocardial injection of autologous bone marrow progenitor cells in patients with ischaemic cardiomyopathy induces improved regional function and reverse remodelling 3 months and 1 year after cell injection, respectively, without significant increase in ejection fraction measurements [64]. Indeed, reverse remodelling induced by cell therapy cause declines in systolic and diastolic volumes so that ejection fraction increase is obscured.

The report by Wall et al. [65] simulated theoretically the effects of injection of small volume of any material into the myocardial wall of infarcted hearts. The data indicate that a small fractional change (0.5 to 5 %) in myocardium wall volume can alter cardiac mechanics, decreasing wall stresses, without improving global function as measured by stroke volume/end diastolic pressure (SV/PED) [65]. These short-term mechanical effects are dependent on the location of the injection, the fractional volume of material added and its relative stiffness to the local myocardium. Moreover, the global pressure–volume relationships, the SV/VED, and the often-reported cardiac metric ejection fraction can be affected by adding non-contractile material [65].

Although needing more study, these results suggest that specific benefits in cardiac function may be derived by reducing elevated local wall stresses implicated in pathological remodelling of the myocardium after infarct.

Importantly, cardiac pump function mediated by cardiomyocyte replacement must be supported by revascularization of the tissue for functional recovery. Although it is known that angiogenesis and arteriogenesis are both mediated by proliferating endothelial cells and their capacity to branch and connect with each other, it has been observed that the cells of the innate immune system take part in these processes in an unexpected manner.

The following section describes in detail the contribution of monocyte and macrophage (MC/Mϕ) populations in regenerative processes and how they have been recently classified as progenitor cells.

## Monocytes/Macrophages and Regeneration

Monocytes (MC) consist of a heterogeneous multifunctional cellular population that plays important roles in immune defence, inflammation and tissue remodelling [66]. They do so by phagocytosis, antigen processing and presentation, and by cytokine production. Under certain stimuli, circulating monocytes give rise to a variety of tissue-resident macrophages (Mϕ) throughout the body, as well as to specialized cells such as dendritic cells and osteoclasts [67]. Monocytes are known to originate in the bone marrow from a common myeloid progenitor that is shared with neutrophils, and they are then released into the peripheral blood, where they circulate for several days before entering tissues and replenishing the tissue macrophage populations. The morphology of mature monocytes in the peripheral circulation is heterogeneous, and these cells constitute 5–10 % of peripheral blood leukocytes in humans [66].

**Fig. 2:**
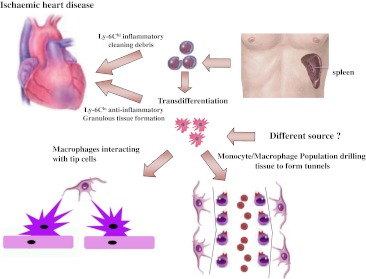
Monocyte/macrophage populations in cardiac repair. Two different populations of monocytes are mobilized to the heart after infarct. The inflammatory monocytes Ly-6C^high^ () are mainly stored in the spleen, while the anti-inflammatory, pro-angiogenic monocytes Ly-6C^low^ () patrol vessels and participate to resolution of inflammation. Transdifferentiation from one type to the other has been reported, although Ly-6C^low^ may occur to the heart from other organs. Macrophage/monocyte populations () have been shown to interact with tip cells eliciting their fusion in developing brain. In addition, they have been shown to drill tunnels into the myocardial tissue to create conduits for the organization of fibro-vascular structures. The origin and phenotype of this specific population has not been analysed.  Smooth muscle cells and  erythrocytes

Studies of the mononuclear phagocyte system, using monoclonal antibodies specific for various cell-surface receptors and differentiation antigens, have shown that there is substantial heterogeneity, which most probably reflects the specialization of individual macrophage populations within their microenvironments. The identification of heterogeneity among peripheral blood monocytes—first, in humans [68], and more recently, in mice [69]—has provided a powerful insight into the nature of myeloid cell subsets and novel ways to assess cell fate and function in vivo. Indeed, analysis of the different populations showed that, in humans, monocytes are present in circulation as CD14^high^CD16^−^ and CD14^low^CD16^+^ cells, enrolled in the tissue during inflammation and chronic disease respectively [70]. In mouse, it is known and well established that monocytes consists of two distinct subsets: the inflammatory cells (Ly-6C^high^) and the Ly-6C^low^ cells, patrolling the resting vasculature, and participating in resolution of inflammation [69] (Fig. 2).

In addition to their pro- and anti-inflammatory response, MC/Mϕ promote tissue repair and regeneration eliciting angiogenesis and cell survival. Indeed, it has been found that monocyte depletion by 5-fluorouracil totally abolishes collateral growth in a model of acute femoral artery ligation in both rabbit and mouse [71]. Studies in rat have shown that Mϕ are present in the tissue as M1 and/or M2 population. M1 Mϕ are associated with muscle necrosis, while M2 population invades muscle once necrotic cells have been removed and is associated with myofiber regenerative [72]. In injured skeletal muscle, partial depletion of the Mϕ population impairs muscle regeneration, whereas reconstitution of bone marrow restores regeneration [70]. Furthermore, it was shown that the function of Mϕ in regenerating skeletal muscle is to support and promote cell growth and to establish cell–cell interaction that protects myogenic precursor cells from apoptosis [70]. Arnold and co-workers showed that injured skeletal muscle recruits MCs exhibiting inflammatory profiles that operate phagocytosis and rapidly convert to anti-inflammatory Mϕ that stimulate myogenesis and fiber growth [70]. In an attempt to provide genetic link between the heterogeneous properties of MC/Mϕ population and their regenerative capacities, it has been shown that CREB-mediated induction of *Cebpb* expression is required in infiltrating macrophages for upregulation of M2-mediated anti-inflammatory cytokine release and muscle regeneration [73].

In addition to their efficiency in re-establishing skeletal muscle morphology and function after injury, MC/Mϕ population has been shown to promote vessel formation after ischaemic damage of the myocardium and in the developing brain. Interestingly, a reduction in the number of vessels containing smooth muscle cells (SMCs) has been observed after macrophage depletion in injured myocardium, indicating that MC/Mϕ are not only regulators of formation of the new blood vessels but also of the subsequent maturation of the newly formed vessels [74]. Indeed, Danenberg showed that macrophage depletion during neointima formation significantly reduced proliferation of SMCs [75]. In different studies, Moldovan and co-authors demonstrated that macrophages drill tunnels in the ischaemic myocardium by activating metalloelastases that digest the extracellular matrix and create conduits for the organization of fibro-vascular structures [76]. Fantin and co-authors, by combining the analysis of mouse mutants defective in macrophage development or VEGF signalling, showed that macrophages promote tip cell fusion, playing a hitherto unidentified and unexpected role as vascular fusion cells [77].

Taken together, these studies show that MC/Mϕ functions relate to their heterogeneous population and have a specific genetic profile. Their properties cannot be summarized uniquely as inflammatory and/or phagocytic but also cover angiogenesis, arteriogenesis and tissue regeneration/remodelling (Fig. 2).

### A Novel Role for Monocyte/Macrophage Populations as Endothelial Progenitor Cells

The concept that monocytes are able to contribute to angiogenesis is not novel. Urbich and colleagues showed that the supposed EPCs have distinct monocytic features and can be cultured from CD14-positive cells [78]. In other studies, De Palma and colleagues showed that a subset of monocytic cells expressing Tie2 and VEGFR2 (Flk1) play a pivotal role in tumour angiogenesis [79, 80]. In a recent report, Kim and co-workers observed that circulating monocytes expressing F4/80, CD31 and VEGFR2 contribute to tumour angiogenesis and revascularization following ischemia [81].

Interestingly, several studies showed that MC/Mϕ elicit angiogenesis and possibly arteriogenesis [71, 82] by releasing pro-angiogenic factors (e.g. agiopoietin, VEGF, bFGF), but also transdifferentiating into various non-phagocytes, such as mesodermal and neuroectodermal lineages [83]. Kuwana and co-authors described a primitive cell population termed monocyte-derived multipotential cells (MOMC) that can differentiate into several distinct mesenchymal cell types, including bone, fat, skeletal and cardiac muscle [83]. MOMCs express several endothelial markers (VE-cadherin, VEGFR1) and are able to uptake acetylated low-density lipoproteins [83]. In a recent publication, it has been shown that human MOMCs incorporated into new forming blood vessels as endothelial cells, indicating that, in a permissive environment, monocytic cells can differentiate into endothelial cells and may represent an autologous source of cells for therapeutic vasculogenesis [84].

The potential of haematopoietic cells to transdifferentiate in endothelial-like cells can be justified by their common origins. Indeed, the concept of crosstalk among the haematopoietic and endothelial lineage is not entirely hypothetical. During embryogenesis, both endothelial and haematopoietic cells derive from a common ancestor, the hemangioblast. Specific environmental factors such as a gradient of FGF expression induce the hemangioblasts to preferentially differentiate towards the endothelial or haematopoietic lineage. In mouse embryo, neovascularization is influenced by monocytes and by their mature derivatives macrophages [82, 85–87], present in the angiogenic fields [88, 89] and preceding the advancement of new capillaries [90]. The possibility that endothelial–haematopoietic signalling occurs in a specific subset of myeloid cells may derive from an ancestral and communal lineage.

Although several lines of evidence support the fact that MC/Mϕ contribute to postnatal vasculogenesis and have a role in angiogenesis following ischaemic myocardial injury, it is not certain if certain MC/Mϕ can become fully functional endothelial cells or if their contribution in angiogenesis is restricted to the production of vascular growth factors. Elucidation of these putative functions is necessary to fully understand the role of monocyte subsets in cardiac repair.

### Monocyte/Macrophage Function in Heart Failure

The function of distinct MC/Mϕ populations in heart repair has been recently investigated [91]. Nahrendorf et al. showed that two distinct subsets of MC/Mϕ participate in myocardial healing after infarct in a sequential manner through phase I (1–3 days) and phase II (4–7 days) [91]. CD11b^high^/Ly-6C^high^ cells exhibiting phagocytic and pro-inflammatory functions, accumulates during phase I and CD11b^high^/Ly-6C^low^ having attenuated inflammatory response and expressing VEGF, are present during phase II [91]. Depletion of phase I, but not phase II, resulted in larger areas of debris and necrotic tissue, indicating that removal of debris requires phase I and its accompanying influx of proteolytic and phagocytic Ly-6C^high^ monocytes immediately after coronary artery ligation. Conversely, the hearts of mice that developed a native Ly-6C^high^ response in phase I, but that received the monocyte inhibitor clodronate liposome in phase II, showed decreased deposition of collagen as well as reduced numbers of microvascular-actin-positive smooth muscle cells and CD31-positive endothelial cells. Thus, granulation tissue formation and remodelling requires non-inflammatory, proangiogenic Ly-6C^low^ monocytes during phase II.

Interestingly, an extramedullary monocyte reservoir in the spleen of mice has been discovered [92]. This monocyte population residing in the spleen of mice is recruited to the site of injury “en masse” as inflammatory and phagocytic cells and represent 41 % of total monocytic population infiltrating cardiac muscle after injury [92]. Thus, spleen stores Ly-6C^high^ monocytes readily recruitable to augment inflammation at distant sites. Moreover, both subsets (Ly-6C^high^ and Ly-6C^low^) exited the spleen in response to MI, yet after 1 day, the ischaemic myocardium recruited Ly-6C^high^ monocytes selectively, suggesting that the excluded Ly-6C^low^ monocytes may have dispersed to other tissues, patrolled the vasculature, or accumulated in the infarct at a later time (Fig. 2) [92].

Finally, Van Amerogen at al. have found that macrophage depletion markedly impaired wound healing, enhancing mortality after myocardial injury [74]. On the same note, lack of MC/Mϕ is associated with earlier development of myocardial dysfunction in hypertensive rat [93]. It is well established that bone marrow mononuclear cells (BM-MNC) increases significantly the percentage of ejection fraction of patients with acute myocardial infarction after intra-coronary infusion (TOPCARE-AMI, BOOST trials) [60, 94]. Although none of these studies determined the effective cell population inducing cardiac function improvement, it is possible to hypothesize that monocytes, being largely represented among BM-MNCs, are the most active cells involved in cardiac repair. One hypothesis is that the presence of monocytes at the sites of vascular or myocardial injury does not represent a catastrophic immune overreaction but a futile attempt at tissue repair [67].

A clear molecular analysis of the cells mobilised at different time points into the injured heart and their recruitment signalling would be important to elucidate the cellular basis of current trials and to inform future therapeutic approaches.

## Concluding Remarks: From an Evolutionary Point of View

**Fig. 3:**
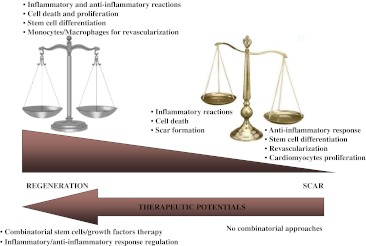
Balancing regenerative processes. Cell therapy is not sufficient to re-establish the molecular and cellular equilibrium leading to function and tissue integrity. Combinatorial approaches must be developed that take into account components of the innate immune system, regulating inflammatory and anti-inflammatory response, as well as new blood vessel formation

Regeneration necessitates of a balanced interplay between different cells and factors recruited to the injured organs (Fig. 3). Resident and/or circulating stem cells and immune cells may both participate in the reconstitution of lost structures. Besides having important roles in orchestrating tissue remodelling, the interplay occurring between the participating cell populations in vertebrate regeneration is unknown. As cardiac regeneration in higher vertebrates is inefficient and scar tissue is formed at expense of cardiac tissue, it appears that fast healing has been selected during evolution of higher mammals at the cost of complete replacement of the lost tissue.

This apparent paradox may have profound implications for the future of clinically relevant heart repair. As Richard Goss wrote “Hence, before any intelligent attempt can be made to restore regeneration where it does not normally take place, it will be necessary to learn what physiological advantages took precedence over regeneration in the course of natural selection” [95]. Accordingly, the advantages of epimorphic regeneration may be outweighed by refinements in the adaptive immune system, overall those that enhanced the acute inflammatory response in wounds [96]. Localized injuries and infections that accompanied the predatory way of life made possible by the development of hinged jaws, evolved with the raise of the cellular components of the adaptive system, allowing greater defences against pathogens in injured tissue, at the costs of tissue’s ability to regenerate perfectly and restore function completely [97]. Indeed, components of the adaptive immune system have been observed to interfere with regeneration [96]. Indeed, regenerating tadpoles have weak cellular and humoral immunity, consisting only of IgM produced by B cells and lacking memory cells [98]. The newt eye environment is built to keep inflammation low during lens regeneration and the peculiar restricted immune response for this organ is associated to the presence of antigen specific regulatory T cells (CD4+ and CD8+) that inhibit the induction and expression of pro-inflammatory cytokines [99].

Although protecting organ integrity and fast healing, scar tissue in mammals can induce pathological outcomes in organs such as the heart and the lung, and over active immune system can cause autoimmune disorders, such as rheumatoid arthritis and scleroderma. In the process of human myocardial regeneration, re-activation of endogenous stem cells or the use of exogenous sources of stem cells for replacing loss of injured cardiomyocytes and endothelial cells (Fig. 3) may not be sufficient to induce regeneration. It may therefore be important to reconsider the role of monocytes and macrophages in the regenerative processes as a population of stem cells actively involved in postnatal vasculogenesis and in angiogenesis during tissue repair. The identification of putative progenitor monocytes and their manipulation in vitro may fuel the field of regenerative medicine with novel approaches. In this context, mobilization of monocyte/macrophage populations is considered a desperate attempt for tissue repair more than a deteriorating immune overreaction.

Therefore, important insights into the loss of regenerative ability during the course of evolution are likely to come from improved understanding of the cellular activities and factors released during inflammation, clarifying the cells and signals leading to regeneration.
